# Carcinogenesis of Triple-Negative Breast Cancer and Sex Steroid Hormones

**DOI:** 10.3390/cancers13112588

**Published:** 2021-05-25

**Authors:** Naoko Honma, Yoko Matsuda, Tetuo Mikami

**Affiliations:** 1 Department of Pathology, Toho University Faculty of Medicine, Tokyo 143-8540, Japan; tetsuo.mikami@med.toho-u.ac.jp; 2 Oncology Pathology, Department of Pathology and Host-Defense, Faculty of Medicine, Kagawa University, Ikenobe 1750-1, Miki-cho, Kita-gun, Kagawa 761-0793, Japan; mazdayoko@gmail.com

**Keywords:** androgen, androgen receptor, estrogen, estrogen receptor-beta, G-protein-coupled estrogen receptor (GPER), sex steroid hormones, triple-negative breast cancer (TNBC)

## Abstract

**Simple Summary:**

Triple-negative breast cancer (TNBC) lacks all of three treatment targets (estrogen receptor-α, ER-α; progesterone receptor, PgR; and human epidermal growth factor receptor 2, HER2) and is usually associated with a poor clinical outcome; however, several sex steroid receptors, such as androgen receptor (AR), ER-β, and G-protein-coupled estrogen receptor, are frequently expressed and their biological and clinical importance has been suggested. Despite the structural similarity between sex steroid hormones (androgens and estrogens) or receptors (AR and ER-β), similar signaling mechanisms of these hormones, and the coexistence of these hormones and their receptors in TNBC in a clinical setting, most studies or reviews focused on only one of these receptors, and rarely reviewed them in a comprehensive way. In this review, the carcinogenic or pathobiological role of sex steroid hormones in TNBC is considered, focusing on common and differing features of hormone actions.

**Abstract:**

Triple-negative breast cancer (TNBC) lacks an effective treatment target and is usually associated with a poor clinical outcome; however, hormone unresponsiveness, which is the most important biological characteristic of TNBC, only means the lack of nuclear estrogenic signaling through the classical estrogen receptor (ER), ER-α. Several sex steroid receptors other than ER-α: androgen receptor (AR), second ER, ER-β, and non-nuclear receptors represented by G-protein-coupled estrogen receptor (GPER), are frequently expressed in TNBC and their biological and clinical importance has been suggested by a large number of studies. Despite the structural similarity between each sex steroid hormone (androgens and estrogens) or each receptor (AR and ER-β), and similarity in the signaling mechanisms of these hormones, most studies or reviews focused on one of these receptors, and rarely reviewed them in a comprehensive way. Considering the coexistence of these hormones and their receptors in TNBC in a clinical setting, a comprehensive viewpoint would be important to correctly understand the association between the carcinogenic mechanism or pathobiology of TNBC and sex steroid hormones. In this review, the carcinogenic or pathobiological role of sex steroid hormones in TNBC is considered, focusing on the common and divergent features of the action of these hormones.

## 1. Introduction

The treatment of breast cancer is primarily involves hormone therapy, anti-human epidermal growth factor receptor 2 (HER2), and chemotherapy [1]. In a clinical setting, the treatment is decided based on the results of pathological examination for estrogen receptor (ER), progesterone receptor (PgR), and HER2 in the tumors (immunohistochemistry for ER and PR; immunohistochemistry and/or in-situ hybridization for HER2) [2,3]. Patients with ER- and/or PgR-positive tumors can be treated with hormone therapy, whereas anti-HER2 therapy is adopted for those with HER2-positive tumors [2,3]. A tumor lacking ER, PgR, and HER2 is called triple-negative breast cancer (TNBC). Patients with TNBC cannot be treated with either hormonal or HER2-targeted therapy, and chemotherapy is usually the only treatment option for them [1,4,5]. Such patients typically have a poor clinical outcome because of the biological aggressiveness of the tumor itself and lack of effective treatment options; one-third of patients with TNBC experience recurrence within the first 3–5 years after diagnosis, whereas others have a good prognosis [4,5,6,7,8]. TNBC has attracted attention because it represents a heterogeneous tumor group with a wide variety of histological, biological, molecular, and/or clinical features. Histologically, apocrine carcinoma, metaplastic carcinoma, medullary carcinoma, and adenoid cystic carcinoma are representative types of TNBC [9]. Extensive molecular studies have been devoted to the subclassification of TNBC, resulting in several subtypes of TNBC, as described below [10].

Hormone (estrogen) unresponsiveness is considered the fundamental characteristic of TNBC; however, studies have revealed the expression of some sex steroid hormone receptors other than classic ER (now renamed ER-α) in TNBC; that is, androgen receptor (AR) [11,12,13,14,15,16,17,18,19,20,21,22,23,24,25,26], second ER (namely ER-β) [27,28,29,30,31,32], or non-nuclear receptors represented by G-protein-coupled estrogen receptor (GPER) [33,34,35,36,37,38]; suggesting the biological and/or clinical relevance of sex steroid hormones in TNBC. Physiologically present androgens and estrogens are made of cholesterol, and commonly have a steroidal structure [39,40,41]. Further, the action of these hormones is exerted through similar mechanisms, not only involving nuclear transcription but also crosstalk with various intracytoplasmic pathways [12,29,33,34]. Physiologically, androgens and estrogens are present in the body, with various rates among organs or individuals. The serum estrogen/androgen ratio is highest in premenopausal women, but lowest in postmenopausal women whose serum estrogen concentration is much lower than in men [42,43,44,45,46,47]. Intra- or peri-tumoral estrogen production is important in ER- and/or PR-positive tumors; however, TNBC or ER- and PgR-negative tumors, where local estrogen production is less active, are directly affected by the serum hormonal status [42,43]. In such a situation, a comprehensive viewpoint on sex steroid hormones is needed; however, despite a large number of studies or reviews on TNBC, most of them focused on the action of either androgens or estrogens in TNBC. In this review, the pathobiological role of sex steroid hormones in TNBC is reviewed, focusing on the common action and divergent role between androgens and estrogens.

## 2. Subclassification of TNBC

TNBC is a biologically heterogeneous tumor entity and not always aggressive. For example, tumors with some special histologies, such as adenoid cystic carcinoma or medullary carcinoma (classical type), are typically classified as TNBC, but are representative of indolent tumors and do not require chemotherapy if node-negative [9,48]. Apocrine carcinoma and invasive lobular carcinoma (pleomorphic type) have attracted interest because of their AR positivity [9,49].

Beyond histology, much effort has been devoted to further classify TNBC. Molecular studies have shown that TNBC can be largely classified into the following categories: (1) Luminal AR (LAR), characterized by AR expression; (2) Immunomodulatory (IM), characterized by an active immune response; (3) Basal-like 1 (BL1), characterized by BRCA mutation; (4) Basal-like 2 (BL2), characterized by the expression of myoepithelial markers such as epidermal growth factor receptor (EGFR) or cytokeratin 5/6 (CK5/6); and (5) Mesenchymal/mesenchymal stem-like (M/MSL), characterized by epithelial-mesenchymal transition represented by E-cadherin negativity [10]. This subclassification is useful, because each subtype not only represents some molecular characteristics, but also suggests dedicated treatment options: AR-targeted therapy for LAR, PARP inhibitors for BL1, mTOR inhibitors for BL2 and M, PD-1/PD-L1 inhibitors for IM, and PI3K inhibitors for M/MSL [10]. In clinical practice, pathological examination including estimation of tumor-infiltrating lymphocytes (TILs) or immunohistochemistry for AR, gross cystic disease fluid protein 15 (GCDFP-15), EGFR, CK5/6, E-cadherin, or PD-L1 is expected as a substitute for molecular analysis [12,50,51,52,53,54,55].

## 3. Sex Steroid Hormones

**Figure 1:**
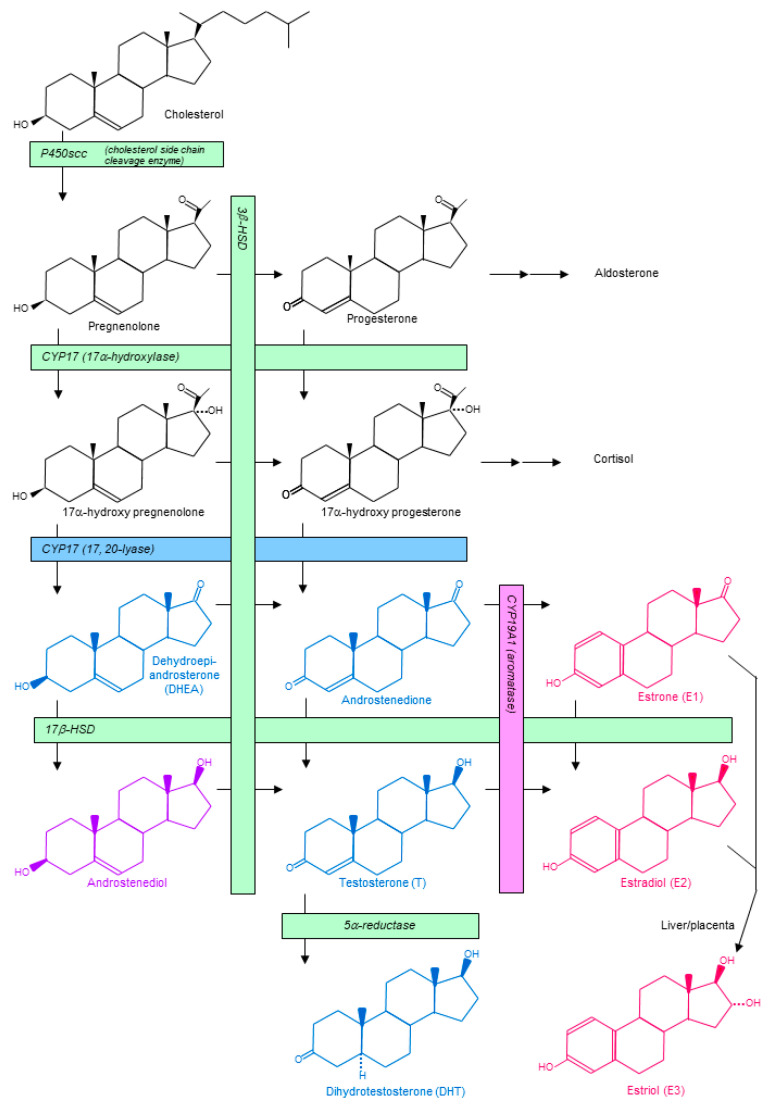
Biosynthesis of sex steroid hormones. Cholesterol is converted to pregnenolone, which is further converted to DHEA or other androgens by CYP17, 3β-HSD, or 17β-HSD, and finally converted to the strongest androgen, DHT, by 5α-reductase. Androgens (androstenedione and T) are converted to estrogens (E1 and E2, respectively) by CYP19A1 (aromatase). Androgens are drawn in blue, while estrogens are in pink. Androstenediol is a constitutive androgen; however, it has a weak estrogen activity (drawn in purple). DHEA, dehydroepiandrosterone; DHT, dihydrotestosterone; E1, estrone; E2, estradiol; E3, estriol; HSD, hydroxysteroid dehydrogenase; T, testosterone.

Sex steroid hormones are largely composed of androgens and estrogens. Generally speaking, androgens are produced from cholesterol with P450scc and 17CYP (17α-hydroxylase and 17, 20-lyase), and they are converted into estrogens by CYP19A1 (aromatase) (Figure 1) [39,40,41]. Physiologically, testosterone (T) and dihydrotestosterone (DHT) are representative of androgens, whereas estradiol (E2), estrone (E1), and estriol (E3) are for estrogens, with each produced from or metabolized into others by various metabolizing enzymes (Figure 1) [39,40,41].

Each of these hormones has its own affinity for its specific receptor, and exerts its functions [40,56]. DHT and E2 are the most potent naturally occurring androgen and estrogen with the highest binding affinity for AR and ER-α, respectively [40,56]. As sex steroid hormones show constitutive similarity, some of them can bind to receptors other than their specific receptor. For example, androstenediol, which constitutively belongs to androgens, can weakly bind to ERs, reportedly with higher affinity for ER-β than ER-α [56].

There are many exogenous substances that exert agonistic or antagonistic effects on sex steroid hormone receptors (Table 1). Isoflavones such as daidzein and genistein are representative phytoestrogens, and act as weak estrogens with higher affinity for ER-β than ER-α. Liquiritigenin, one of the flavanones, acts as a selective agonist for ER-β, but has a partial agonistic effect on ER-α. Prinaberel (ERB-041) and diarylpropionitrile (DPN) are representative synthetic ER-β selective agonists: >200- and >70-fold selectivity for ER-β over ER-α, respectively [57]. GPER selective ligands include the agonist G1 and antagonists G15 and G36 [58,59]. Many other synthetic agents act on sex steroid hormone receptors and modulate their function, some of which are adopted as standardized endocrine therapy for breast cancer or prostatic cancer, as shown below (Section 5).

## 4. Sex Steroid Hormone Receptors Other Than ER-α and PgR

Although TNBC is defined as negative for both ER-α and PgR, some TNBC express other sex steroid hormone receptors: AR [11,12,13,14,15,16,17,18,19,20,21,22,23,24,25], ER-β [27,28,29,30,31,32], or GPER [33,34,35,36,37,38]. AR and ER-β belong to the ER type subgroup of the nuclear receptor superfamily, and typically transmit genomic signaling; however, the existence of non-genomic signaling pathways through these receptors or non-nuclear receptors located in the cytoplasmic membrane or cytoplasm has been reported and attracted attention. Genomic signaling through nuclear receptors needs 12–28 h, whereas non-genomic signaling through membrane-bound receptors needs seconds to 120 min. As described below, sex steroid hormones exert their functions through the integrated action of genomic and non-genomic signaling. This mechanism is evolutionarily conserved [60], and is also present in progesterone signaling [61]. The nuclear and non-nuclear receptors are separately reviewed here.

### 4.1. Nuclear Receptors (ER-β and AR)

**Figure 2:**
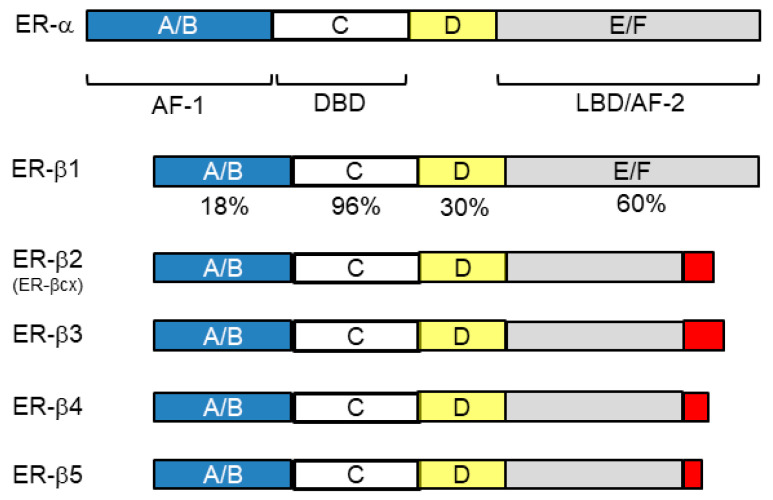
Structure of nuclear receptors, ER-α, ER-β1 (wild-type of ER-β), and ERβ isoforms (ER-β2-5). The nuclear receptor superfamily is structurally common, having the following domains: A/B (activation function, AF-1), C (DNA-binding domain, DBD), D (hinge region), E/F (ligand-binding domain, LBD/AF-2). The C domain is most homologous among family members. ER-β1 shares 96 and 60% homology with ER-α at DBD and LBD, respectively. In ER-β, LBD is coded by alternatively spliced exon 8 of ESR2 (ER-β-coding gene), resulting in five different forms of ER-β: ER-β1 (wild-type) to ER-β5.

The nuclear receptor superfamily is structurally common, and all have the following domains: A/B (activation function, AF-1), C (DNA-binding domain, DBD), D (hinge region), and E/F (ligand-binding domain, LBD/AF-2) (Figure 2) [29,62]. Among these domains, the C domain is most homologous among family members, with two zinc finger motifs. AF-1 has the ability to activate transcription independent of a ligand, whereas the action of AF-2 is ligand-dependent and regulates the action of AF-1.

**Figure 3:**
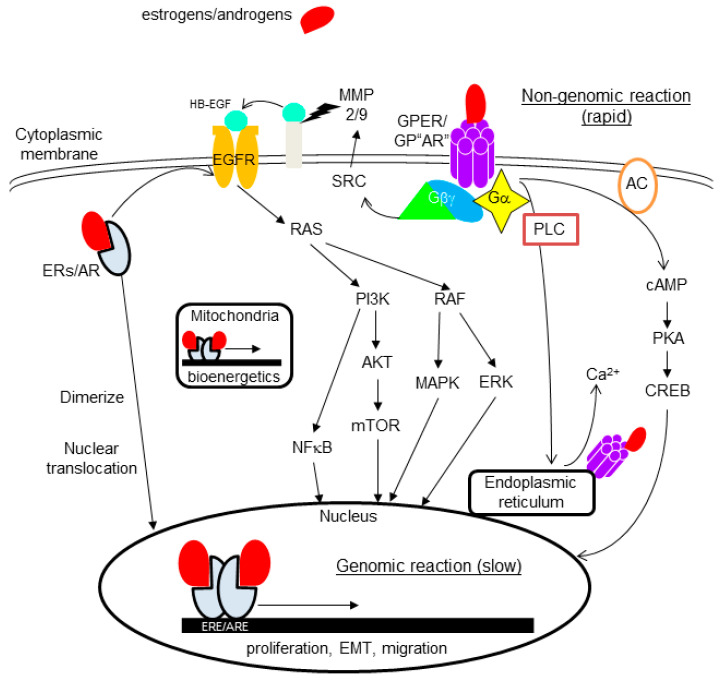
Actions of androgens and estrogens. Both hormones exert a genomic reaction (slow) through their specific nuclear receptors, AR or ERs. In an inactive form, these receptors are located in the cytoplasm. Upon ligand binding, these dimerize and translocate to the nucleus, bind to each hormone response element (HRE: ARE for AR and ERE for ERs) of the targeted genes, and activate their transcription. A non-genomic reaction (rapid) is exerted through non-nuclear receptors, represented by G-protein-coupled estrogen receptor (GPER). GPER/GP“AR” activated by each ligand causes various reactions, including pathways such as: (1) cAMP production through adenylyl cyclase (AC) leading to PKA/CREB activation; (2) mobilization of calcium from the endoplasmic reticulum through phospholipase C (PLC); (3) activation of SRC proteins, promoting the activation of MMP-2/9, resulting in EGFR transactivation, which lead to the activation of MAPK, ERK1/2, PI3K/Akt/mTOR, or NFκB. GPER is also present in the endoplasmic reticulum or nucleus. ER-β, frequently observed in mitochondria, is important in bioenergetics.

In an inactive form, these receptors localize to the cytoplasm, but upon ligand binding, dimerize, translocate to the nucleus, bind to a hormone response element (HRE: ARE for AR and ERE for ERs) of the targeted genes, and activate their transcription, finally transmitting androgenic or estrogenic genomic signaling (Figure 3) [12,63]. Each ligand directly activates the transcription of primary responsive genes (for example, PgR, prolactin, oxytocin, c-fos, or pS2 for estrogen-ER-α binding). Products of primary responsive genes further promote the transcription of secondary or tertiary responsive genes, finally resulting in a dynamic action. These signal pathways crosstalk with the other signal transduction pathways through growth factor receptors or various membrane-bound receptors (Figure 3) [12,63,64].

As described above, ER-α, ER-β, and AR have many similarities in their structure or action pattern, but of course have their own characteristics. Below are the characteristics of ER-β compared with ER-α: ER- β shares 96 and 60% homology with ER-α at DBD and LBD, respectively, suggesting the ability of binding to similar DNA sites, and both similar and distinct ligand preferences (Figure 2). LBD is coded by alternatively spliced exon 8 of ESR2 (ER-β-coding gene), resulting in five different forms of ER-β: ER-β1 to ER-β5 [29,62,65]. ER-β1, the wild-type, can bind to ligands; however, ERβ2-5 variants, with a truncated form of this domain (Figure 2), lack binding ability but can dimerize with other ERs, which enables these ERβ variants to dominant negatively regulate estrogen signaling [64]. With less homology with ER-α, LBD represents the characteristics of ER-β; however, the presence of variant forms, and the lack of robust antibodies for each ER-β variant, complicates the biological and clinical importance of ER-β specifically in breast cancer [29,66]. Generally speaking, ER-β has a weaker transcription ability than ER-α, and if co-expressed with ER-α, ER-β negatively regulates the function of ER-α [56]. Compared with ER-α, which distributes most abundantly in female reproductive organs such as the breast and uterus, ER-β widely distributes through systemic organs irrespective of sex. Studies of nuclear ERs in other species revealed that some species have ER-β but not ER-α, and ER-β is more potent than ER-α to transmit estrogenic actions [67]. These observations suggest that ER-β is evolutionarily more primitive and is physiologically more fundamental than ER-α. Furthermore, in breast tissue, the presence of ER-β is observed more widely than ER-α regardless of malignancy; that is, ER-β is present in normal epithelium, mesenchymal tissue, cancer stem cells, and even in some TNBC [27,29,64,68].

### 4.2. Non-Nuclear-Located Sex Steroid Hormone Receptors

The presence and importance of non-nuclear-located sex steroid hormone receptors have been suggested from the finding of rapid actions induced by estrogens or androgens on various type of cells or tissues, which occurs within minutes after stimulation [33,34,69]. The underlying mechanism behind the rapid action has been intensively studied, and proved to be through specific kinases and modulation of a significant number of cellular processes (Figure 3). 

#### 4.2.1. Membrane-Bound Receptors

As membrane receptors, the presence and importance of G-protein-coupled receptor (GPCR) for estrogens and androgens have been extensively studied. Members of the GPCR family, with seven-transmembrane receptors, typically locate on plasma membranes, and transmit extracellular signals to cells, but have also been shown to locate in the endoplasmic reticulum or nucleus. GPER, formerly known as GPR30, is the most representative and the most studied membrane-bound sex steroid hormone receptor. GPER activated by ligand binding causes various reactions, including pathways such as: (1) cAMP production leading to PKA/CREB activation; (2) mobilization of calcium from the endoplasmic reticulum through activation of phospholipase C (PLC); (3) activation of SRC proteins promoting the activation of MMP-2/9, resulting in EGFR transactivation, leading to the activation of MAPK, ERK1/2, PI3K/Akt/mTOR, and NFκB (Figure 3), etc. [33,34]. The presence of GPCR for androgens (GP“AR”) has long been suggested, and recently, GPRC6A, ZIP9, and OXER1 were proved to have the ability to act as membrane androgen receptors [12,69].

ERs and AR, classically known as nuclear receptors, also locate on the cytoplasmic membrane, and transmit rapid non-genomic signaling through mechanisms resembling those of GPER (fluctuation of cAMP and Ca^2+^, or stimulation of protein kinase pathways, etc.) [70].

Crosstalk between those membrane receptor signaling processes and other signal-transduction pathways, such as the EGFR and insulin-like growth factor 1 receptor-signaling pathways, has been suggested to be important in the carcinogenic mechanism; however, the pathobiological role of those membrane receptors remains unclear in breast cancer as well as other malignancies [12,64].

#### 4.2.2. Cytoplasmic Receptors

**Figure 4:**
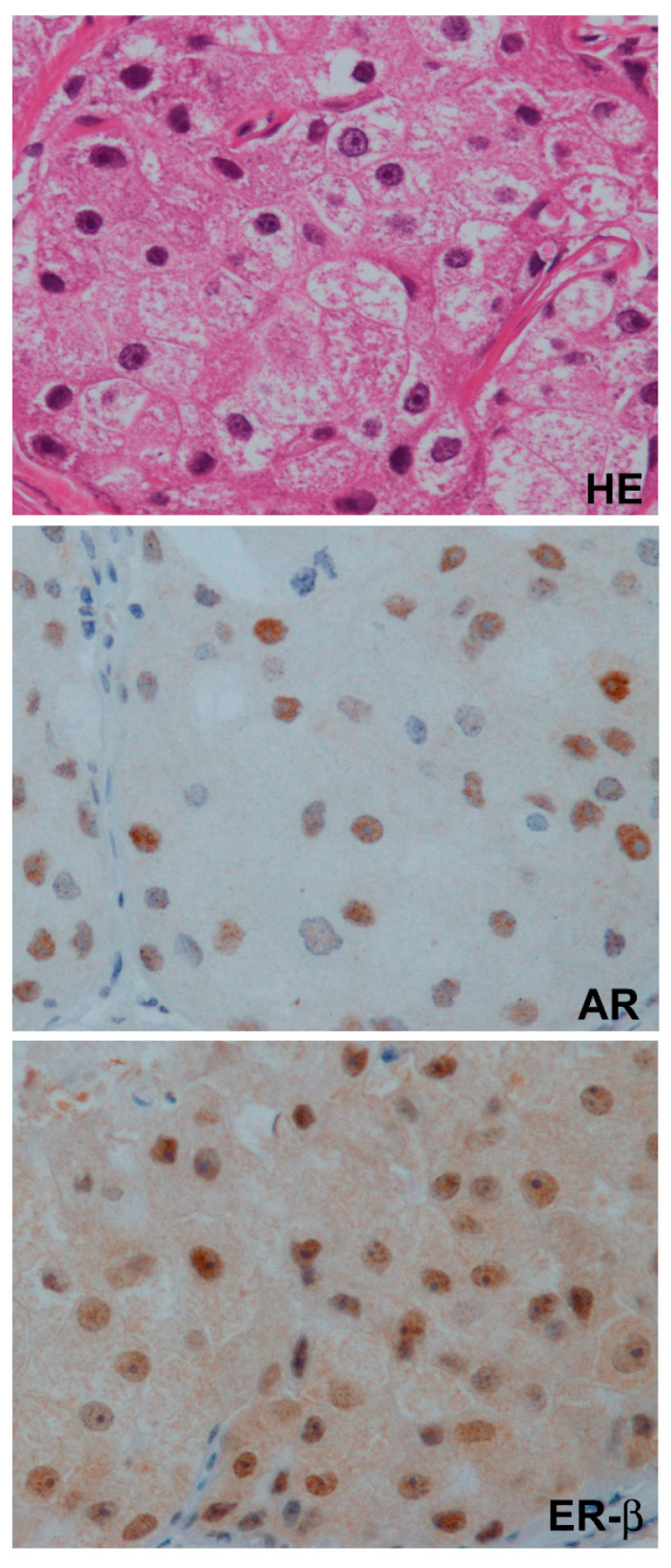
A case of apocrine carcinoma co-expressing nuclear androgen receptor (AR) and estrogen receptor-β (ER-β). Cytoplasmic staining is relatively stronger for ER-β than AR. Hematoxylin-eosin staining, HE.

There is evidence for cytoplasm-located sex steroid hormone receptors in breast pathology. GPER is detectable not only in the cytoplasmic membrane but also in endoplasmic reticulum [33]. Among nuclear receptors (ER-α, ER-β, AR), relatively frequent and intense cytoplasmic staining has been reported for ER-β in many immunohistochemical studies, suggesting that a non-genomic action through cytoplasmic receptors may be more important in ER-β than others (Figure 4) [30,64,71,72]. Studies showed ER-β localization in mitochondria, and its importance in bioenergetics [73]. Mitochondrial ER-β putatively exerts its function on mitochondrial DNA-encoded genes through an ERE-like sequence (Figure 3) [29].

## 5. Agents Inhibiting the Effect of Sex Steroid Hormones

**Figure 5:**
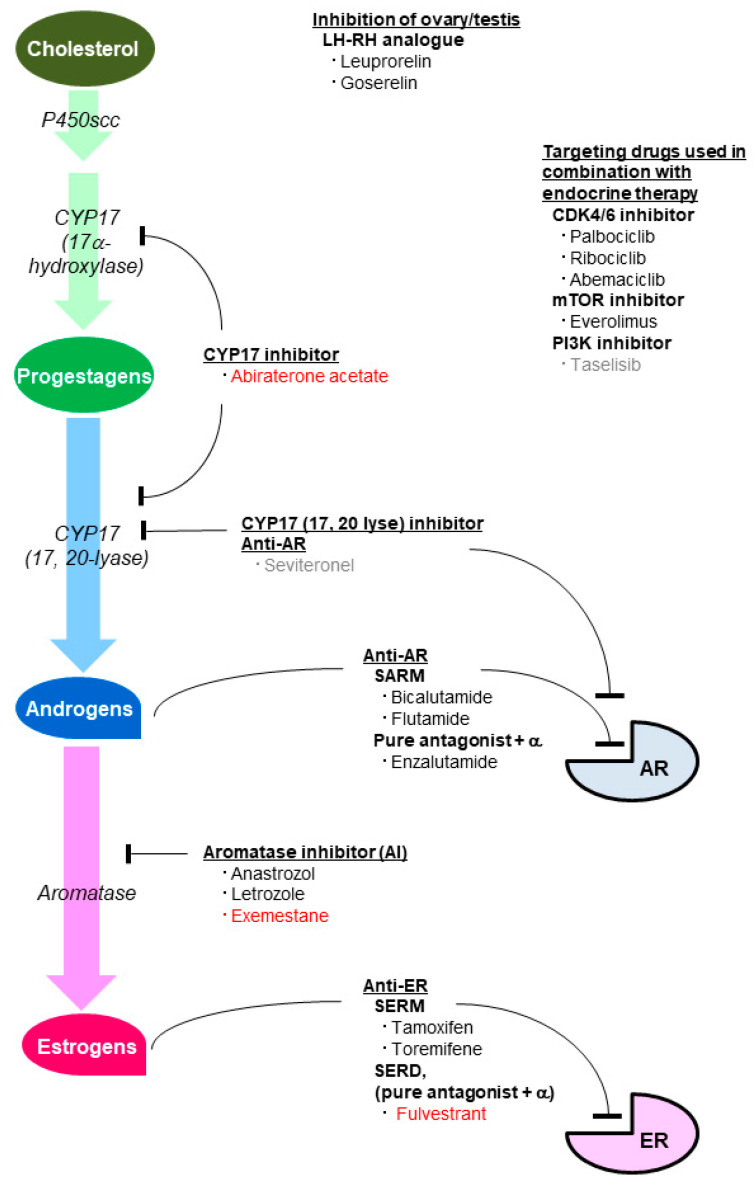
Therapies inhibiting sex steroid hormones and molecular targeting drugs used in combination with endocrine therapies. Androgen-inhibiting therapies, such as anti-AR therapies and CYP17 inhibitors, are standardized for prostatic cancer, whereas estrogen-inhibiting therapies, such as anti-ER therapies and AI, are for ER/PgR-positive breast cancer (AI, for postmenopausal women). LH-RH analogues are used for both prostatic cancer and premenopausal ER/PgR-positive breast cancer. Steroidal drugs are shown in red words. All agents except seviteronel and taselisib are currently available in clinical practice. Some of the drugs are expected to be effective for patients with TNBC (for example, androgen-inhibiting therapy for AR-positive TNBC, or tamoxifen for ER-β-positive TNBC). AI, aromatase inhibitor; AR, androgen receptor; ER, estrogen receptor; PgR, progesterone receptor; SARM, selective AR modulator; SERD, selective ER downregulator; SERM, selective ER modulator. Underlines, functional categories.

Agents inhibiting the effect of estrogens and androgens are now routinely used to treat hormone receptor (ER-α and/or PgR)-positive breast cancer and prostatic cancer, respectively (Figure 5); however, accumulating preclinical and clinical studies have shown that some of them are promising as treatments against TNBCs. Targeting strategies against estrogens and androgens have many similarities, and they can be summarized in a comparative way as below.

### 5.1. Agents Inhibiting the Estrogenic Effect

Several estrogen-inhibiting therapies are currently available for ER-α/PgR-positive breast cancer, which are largely divided into two categories: anti-ER therapy and therapy inhibiting estrogen production (Figure 5) [1]. Regarding anti-ER therapy, tamoxifen and toremifene are nonsteroidal selective ER modulators (SERM) with a partial agonistic effect on ER. Fulvestrant is a selective ER downregulator (SERD), which has the combined action of a pure antagonistic effect and ER-degrading effect. SERM and SERD are applicable regardless of the menopausal status. Interestingly, SERM and SERD were shown to be the GPER agonists [74], and the expression of GPER has been suggested to be associated with tamoxifen resistance [75,76,77,78,79]. LH-RH analogues such as leuprorelin and goserelin downregulate estrogen production in the ovary, and are used for premenopausal patients. Aromatase inhibitors (AI), which downregulate peripheral estrogen production, are used for postmenopausal patients [1]. Exemestane is a steroidal AI, whereas anastrozole and letrozole are nonsteroidal AI. Metroxyprogesterone acetate (MPA), a first-generation progestin and a PgR agonist, is another treatment option; however, MPA also has binding affinity for other steroid receptors such as AR, and the anti-tumor mechanism in breast cancer is still unclear. All agents except LH-RH agonists and fulvestrant are orally available.

Activation of the PI3K-AKT-mTOR pathway or cell-cycle promoter CDK4/6 has been shown to be important as the mechanism of how breast cancer acquires resistance to endocrine therapy. Dual inhibition of estrogen with mTOR (everolimus) or CDK4/6 (palbociclib, abemaciclib) is now an option for recurrent/metastatic disease [80,81,82].

### 5.2. Agents Inhibiting the Androgenic Effect

At present, several therapies inhibiting the androgenic effect have been standardized for prostatic cancer. Androgen-inhibiting therapies, like estrogen inhibiting therapies for breast cancer, are largely divided into two categories: anti-AR therapies and therapies inhibiting androgen production (Figure 5) [83,84]. Bicalutamide and flutamide, orally available nonsteroidal competitive AR inhibitors, act as selective androgen receptor modulators (SARM). They are similar to tamoxifen having a partial agonistic effect on their specific receptor. Enzalutamide is a pure AR antagonist as well as an inhibitor of AR nuclear translocation, DNA binding, and coactivator mobilization. Enzalutamide resembles fulvestrant, having the combined action of a pure antagonistic effect and other effects. LH-RH analogues, which downregulate androgen production in the testis, are also used to treat prostatic cancer. Abiraterone acetate is a potent, orally available, steroidal selective inhibitor of both 17α-hydroxylase and 17, 20-lyase, which targets adrenal and tumor intracrine androgen biosynthesis. Abiraterone acetate resembles exemestane regarding its function and steroidal nature. Seviteronel, still unstandardized even in prostatic cancer treatment, is an oral, non-steroidal 17, 20-lyase inhibitor and AR antagonist [85]. Seviteronel, unlike abiraterone acetate, is free from the side-effect of inhibiting cortisol production (Figure 1 and Figure 5).

## 6. The Role of Sex Steroid Hormones in TNBC in a Preclinical Setting

The role of sex steroid hormones in TNBC has been extensively studied in a preclinical setting. A large number of studies used TNBC cell lines, each of which has a characteristic feature regarding the expression pattern of AR or ER-β, and examined the effect of suspected agonists or antagonists, including agents used in endocrine therapy for prostatic or breast cancer.

### 6.1. The Role of AR in TNBC

The androgen-signaling pathway has been suggested to play a role in breast cancer pathogenesis, although both stimulatory and inhibitory effects have been indicated [12,86]. Conflicting results can be at least partly attributed to the underlying molecular phenotype or co-expression of hormone receptors other than AR. Recently, studies on TNBC cell lines expressing AR (molecularly the LAR subtype), such as MDA-MB-453, SUM185PE, CAL-148, and MFM-223, have accumulated. As expected, these cells are not affected by estrogens or anti-estrogens, and androgens exert a proliferative effect, whereas AR siRNA or an AR-inhibitor (flutamide, bicalutamide, and enzalutamide) exerts a suppressive effect on these cells, suggesting an AR-dependent mechanism of tumor growth; hence, the possibility of AR-targeting therapy for LAR [87,88]. The underlying mechanism has been attributed to the participation of decreased apoptosis, cell-cycle regulation, or crosstalk with other pathways such as the PI3K-AKT-mTOR pathway [87,88]. AR-positive TNBC was shown to frequently have activating mutations in the phosphatidylinositol-4, 5-biphospate 3-kinase catalytic subunit alpha (PIK3CA) or pAKT, suggesting a tumor-promoting effect, but it was sensitive to combined inhibition by PI3K and AR [89]. In contrast, PTEN, which negatively regulates the PI3K-AKT-mTOR pathway, was reportedly up-regulated by AR expression in some studies, suggesting the inhibitory effect of AR in TNBC [12].

AR activation has also been suggested to be associated with migration, invasiveness, and metastasis of tumor cells. Zinc-finger enhancer binding protein (ZEB1) suppresses the expression of E-cadherin, a cell adhesion molecule, and promotes epithelial-to-mesenchymal transition (EMT). Graham et al. showed that ZEB1 and AR regulate each other to promote cell migration or EMT in TNBC cell lines (MDA-MB-231 and MDA-MB-435), indicating a suppressive effect of bicalutamide on ZEB1 [90]. Extracellular matrix degradation is an important process in tumor growth and angiogenesis. AR was also shown to induce the expression of metalloproteinase (MMP), particularly MMP2 or MMP9, suggesting a role in EMT [88]. Giovannelli et al. showed that androgen activation of Src/PI3K signaling drives the invasiveness of TNBC cells (MDA-MB-231 and MDA-MB-453) [91].

Like dual inhibition of estrogen and mTOR or CDK4/6 in recurrent/metastatic ER-α/PgR-positive breast cancer, the possibility of dual inhibition of androgen and mTOR or CDK4/6 is now attracting interest in TNBC. The combination of palbociclib with enzalutamide reportedly showed in-vitro activity in RB-proficient and AR-positive TNBC [92]. Christenson et al. showed the combined effect of seviteronel and abemaciclib in AR-positive TNBC [93]. Gordon et al. showed that combining an AR antagonist and everolimus resulted in the synergistic inhibition of proliferation [94].

### 6.2. The Role of ER-β in TNBC

In ER-α-positive breast cancer, ER-β negatively regulates the proliferative effect of ER-α [56]. In TNBC, the role of ER-β has been extensively studied in TNBC cell lines such as MDA-MB-468, MDA-MB-231, Hs578T, and HCC1806. A large number of studies indicated an inhibitory effect on proliferation, as follows [29]. Exogenous or ectopic expression of ER-β1 in TNBC cell lines inhibited cellular proliferation, and this inhibitory effect was promoted by an ER-β 1 agonist or suppressed by an ER-β 1 antagonist, suggesting ER-β 1 ligand-dependent activity for TNBC suppression. Cell-cycle analysis revealed that the anti-proliferative effect of ER-β 1 is largely through G1 cell arrest [95], which is attributed to the ER-β-mediated downregulation of genes involved in cell-cycle progression, including some cyclin-dependent kinases (CDK), such as CDK1, CDK7, and the cyclins B and H.

A suppressive role of ER-β in invasiveness or metastasis of TNBC has also been suggested. So far, several mechanisms have been proposed for the ER-β-mediated inhibition of metastasis or EMT [29]. (1) Ligand-activated ER-β promotes the production of cystatins, which block the TGF-β/SMAD pathway driving invasiveness, cell migration, and metastasis formation [96]. (2) ER-β blocks EMT through the inhibition of P53 mutant proteins [97]. (3) ER-β destabilizes EGFR, resulting in the upregulation of miR-200a/b/429, which leads to ZEB1 repression [98]. (4) Crosstalk between ER-β and AR, which will be detailed in the next section. This indicates the possibility of ER-β-stimulating therapy for ER-β-positive TNBC.

Recently, Yan et al. compared the biological role of ER-β 1, ER-β 2, and ER-β 5 in MDA-MB-231 cells, by up- or downregulating them [99]. They showed that ER-β2/ER-β5 is associated with cellular proliferation, migration, invasion, and proto-oncogene survivin increase, whereas ER-β1 has the opposite effect [99]; this is consistent with the clinical data indicating that ER-β1 is a predictor of a favorable outcome whereas ER-β2/ER-β5 predicts an unfavorable one [27,100].

Mukhopadhyay et al. showed that ER-β interaction with wild-type and mutant TP53 had a pro-proliferative and anti-proliferative effect, respectively, in breast cancer cell lines (including TNBC cells), suggesting the importance of the TP53 status as a determinant of the tumorigenic role of ER-β [101]. They also showed that tamoxifen increased ER-β-mutant TP53 interaction, causing TP73 reactivation and apoptosis [101]. The predictive value of ER-β for the chemotherapy response was also suggested in cancers with defective P53 but not in those with wild-type P53 in another study [102]. The TP53 status may be a key factor to determine the role of ER-β in the pathobiology of or therapy for TNBC.

There have been in-vitro studies to develop a novel therapeutic strategy for TNBC modulating the ER-β function. Schϋler-Toprak et al. showed that the invasiveness of MDA-MB-231 and HS578T TNBC cells decreased with ER-β agonists ERB-041 and WAY200070, whereas the agonists liquiritigenin and 5α-androstane-3β, 17β -diol only reduced invasion of MDA-MB-231 cells. In contrast, knockdown of ER-β by siRNA transfection increased the invasiveness of MDA-MB-231 cells through activating TGFβ signaling or inducing the expression of a network of genes promoting invasion [103]. Furthermore, in an in-vitro model examining bone-directed invasion, liquitrigenin and ERB-041 reduced the invasiveness of ER-β-positive TNBC cell lines HCC1806 and HCC1937, suggesting the possibility of using an ER-β agonist to inhibit bone metastasis [104].

As shown above, most studies indicated the suppressive role of ER-β in TNBC, specifically, with mutant TP53; however, Ma et al. recently showed the absence of ER-α and upregulation of ER-β in breast cancer stem cells (BSCs). They showed that ER-β is responsible for the proliferative role of estrogens in BSCs, and that a selective inhibitor of ER-β (PHTPP) blocks the proliferation of patient-derived BSCs (irrespective of luminal or TNBC), suggesting the possibility of using an ER-β inhibitor as a therapeutic strategy against BSCs [68].

### 6.3. Crosstalk between AR and ER-β

**Figure 6:**
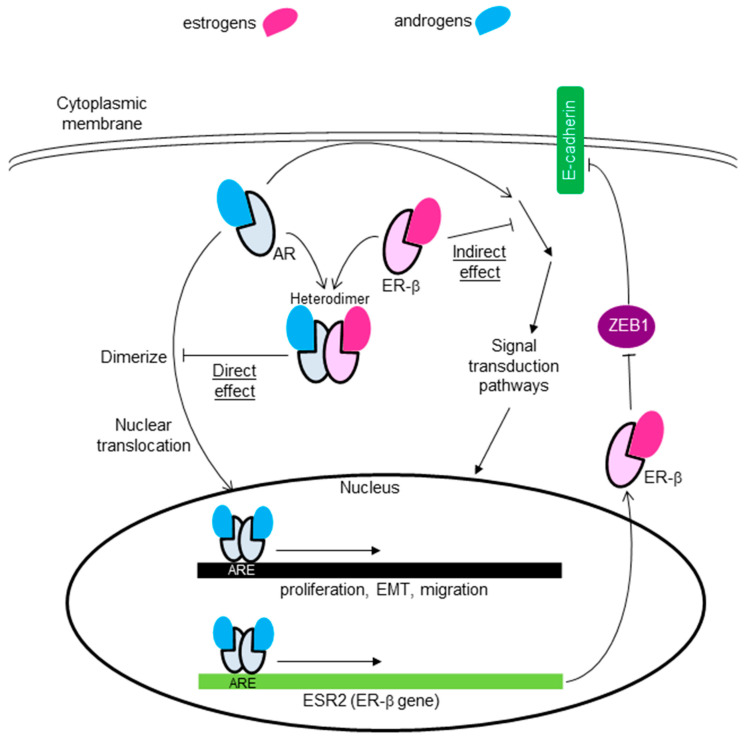
Proposed mechanism for the combined effect of AR and ER-β on TNBC. ER-β expression reverses the aggravating role of AR: indirectly through inhibition of the signal transduction pathway activated by AR, or directly by forming a heterodimer with AR, inhibiting it from forming homodimers [105]. ER-β1 also suppresses the invasion, migration, and metastatic abilities of these cells by suppressing ZEB1. Activation of AR promotes ER-β production by functioning as a transcription factor that directly binds to the ER-β promoter [106].

Some studies indicated the combined effect of AR and ER-β or GPER in TNBC cell lines. Anestis et al. examined the effect of ER-β expression on MDA-MB453 AR-positive TNBC cells, and showed that ER-β expression reversed the aggravating role of AR: indirectly through the inhibition of the PI3K/AKT pathway activated by AR, and directly by forming a heterodimer with AR, preventing it from forming homodimers (Figure 6) [105]. They also showed that ER-β expression increased the sensitivity of MDA-MB453 cells to enzalutamide, suggesting that the co-expression of AR and ER-β is a predictor of the usefulness of anti-androgen therapy [105]. Song et al. generated stable ER-β1-expressing AR-positive TNBC cell lines (MDA-MB-231 and Hs578T), and showed that ER-β1 suppressed the invasion, migration, and metastatic abilities of these cells by suppressing ZEB1. They also showed that activation of AR increased the anti-metastatic effect of ER-β in these cells by functioning as a transcription factor that directly binds to the ER-β promoter (Figure 6) [106]. McNamara et al. also reported androgen-dependent upregulation of ER-β in a subset of AR-positive TNBC cell lines [107].

### 6.4. The Role of Non-Nuclear-Located Receptors in TNBC

The presence and importance of non-nuclear-located sex steroid hormone receptors have also been suggested in TNBC. For example, a rapid estrogenic or androgenic action has been reported in TNBC cell lines lacking specific nuclear receptors (ER-α/ER-β and AR) [34,69]. Despite a significant number of studies, the role of non-nuclear-located receptors in TNBC is largely unknown. Even for GPER, which is the most studied among them, its pathobiological role in TNBC is unclear [34,108]. Each study suggesting the proliferative or suppressive role of GPER in TNBC cell lines showed the mechanisms of how GPER plays biological roles, which include a wide range of carcinogenic hallmarks such as disruptions of cell proliferation, the cell cycle, EMT, and angiogenesis. Controversy may at least be partly attributed to the ligand used (estradiol, tamoxifen, or G1); however, more studies are needed to clarify its role in TNBC [108]. The role of membrane AR in TNBC is mostly unknown, because it is only recently that candidates for membrane AR were indicated [69]. Shen et al. reported that activation of AR suppressed GPER expression, and promote TNBC cell growth. They further indicated that AR suppressed GPER by binding directly to the promoter of GPER [109].

## 7. The Role of Each Sex Steroid Hormone in TNBC in a Clinical Setting

In a clinical setting, the role of each sex steroid hormone in TNBC has been mainly investigated through its immunohistochemically detected receptors.

### 7.1. The Role of AR in a Clinical Setting

AR expression is frequently observed in a subset of TNBC, which is classified as LAR. The most typical histological feature of LAR is an apocrine morphology, but AR expression is not limited to tumors with an apocrine morphology. In a recent systematic review by Xu et al., the reported AR-positivity rates in TNBC ranged from 12–59% (28% in total) [110]. TNBC with an apocrine morphology or AR-positivity is reportedly frequent in older patients, and we recently reported that the rate of AR-positivity and apocrine morphology was 65 and 43%, respectively, in TNBC from patients ≥ 75 [111]. The prognostic importance of AR in TNBC has been examined in a large number of studies; however, the results were controversial. In some studies, AR-positivity was related to a favorable prognosis, whereas opposite results were reported in others [12,13,16,17,24]. In the meta-analysis by Xu et al. including 27 studies involving 4914 patients with TNBC, AR expression was not associated with any prognostic factors (disease-free survival, overall survival, distant disease-free survival, or recurrence-free survival) [110]. We showed that AR-positivity was related to a favorable prognosis in patients aged 75 or over, but not in those aged 55–64, which may at least partly explain the controversial results regarding the prognostic importance of AR in TNBC [111]. This finding, along with the higher-AR positivity rate in older patients, may indicate the more important role of androgens and AR in the pathogenesis of TNBC in this population. TNBC diagnosed at an older age mostly develops after menopause. In such a condition, cells adjusted for the relative dominance of androgens over estrogens may survive and proliferate, finally forming AR-positive TNBC.

Graham TR et al. showed that ZEB1 and AR were co-expressed in a majority of TNBC in a clinical setting (70% for ZEB1, 67% for AR). Along with experimental data, they suggested the inhibitory effect of androgen-targeting therapy against cell migration through suppression of ZEB1 [90].

Lehmann et al. showed that AR-positive TNBC frequently showed activating mutations in PIK3CA or pAKT, suggesting a tumor-promoting effect, but the possibility of the dual inhibition of PI3K and AR as a treatment strategy [89].

### 7.2. The Role of ER-β in a Clinical Setting

The prognostic value of ER-β in TNBC has been examined in a large number of studies, but the results are conflicting, which has been attributed to the differences of: (1) type of ER-β examined (ER-β1 to ER-β5); (2) intracellular component estimated (nuclear staining or cytoplasmic staining); (3) antibody used in immunohistochemistry; (4) population of involved patients (age, stage, type of systemic therapy); (5) cancer cell type, cancer stem cells or differentiated cancer cells; (6) TP53 status (wild or mutant). Despite divergent results, a favorable prognosis associated with ER-β1-positive tumors has been supported by several studies [27,28,32]. Others reported that positivity for another isotype (ER-β5 or cytoplasmic ER-β2) was a predictor of a poor clinical outcome [100,112]. Yan et al. recently showed that ER-β2/ER-β5 were predominantly expressed in TNBC, and were predictors of a poorer outcome [99]. These findings suggest the importance of the absolute amount and relative ratio of these isoforms to stratify TNBC by prognosis or to consider ER-β-targeting therapy. Mukhopadhyay et al. examined the prognostic value of ER-β expression and the TP53 mutation status in a basal-like TNBC subgroup, and showed that the high ER-β expression level in mutant TP53-expressing tumors was associated with a better prognosis, suggesting the importance of a combined consideration of ER-β and the TP53 status to stratify TNBC by prognosis [101]. They also showed an in-vitro study whereby tamoxifen increased ER-β-mutant TP53 interaction, causing TP73 reactivation and apoptosis [101]. Interestingly, some studies, including ours, showed that a favorable prognostic value of ER-β1 is observed in patients treated with tamoxifen [27,29,32]. These findings suggest the promise of further studies to repurpose tamoxifen and evaluate the importance of ER-β examination in clinical treatment for TNBC.

### 7.3. Correlation of AR and ER-β in Clinical TNBC

In a clinical setting, most studies indicated a positive correlation between the expression of AR and ER-α, or ER-β and ER-α, suggesting a positive correlation between expressions of AR and ER-β. Indeed, we showed a positive correlation between AR and ER-β in a study including 403 cases [16]. It is controversial whether this is also true for ER-α-negative tumors or TNBC. In our previous study of 48 apocrine carcinomas, 1 (2%), 29 (60%), and 35 (73%) were positive for ER-α, AR, and ER-β, respectively. Twenty-two of 48 apocrine carcinomas co-expressed AR and ER-β (Figure 4), although expression levels of AR and ER-β were not correlated [72]. Further, we also showed that ER-β1 expression in apocrine carcinomas was negatively correlated with prognostic factors such as the tumor size or grade [71]. Song et al. immunohistochemically examined 82 TNBC clinical samples, and showed that expression of ER-β1 was positively correlated with that of AR or E-cadherin, and negatively with ZEB1, suggesting reduced EMT or aggressiveness for ER-β1-positive tumors [106]. Despite the controversy regarding the correlation between the expression of AR and ER-β in TNBC, it is important to remember that ER-β is co-expressed with AR and may play a suppressive role in a subset of TNBC such as LAR tumors or apocrine carcinomas, suggesting the need for their combined consideration in the treatment of these tumors. Goto et al. examined the combined expression of AR, ER-β, and P53 in metastatic TNBC, and showed that AR-/ER-β+/P53+ was significantly correlated with a poorer outcome [113].

### 7.4. The Role of Non-Nuclear Receptors in a Clinical Setting

So far, GPER has been the most studied non-nuclear receptor in clinical samples of breast cancer. In several reports, GPER expression was associated with tamoxifen resistance in ER-positive tumors. In patients treated with tamoxifen, GPER was negatively correlated with relapse-free survival, or was expressed more in recurrent than primary tumors [76,77]. As for TNBC, Yu et al. reported that GPER expression was prevalent, and was associated with phospho-ERK1/2, a larger tumor size, and more advanced stage, suggesting a tumor-progressive role of GPER in TNBC [36]. In contrast, Chen et al. and Liang et al. reported that GPER expression was negatively associated with a higher grade, stage, or lymph node metastasis, while positively associated with a favorable outcome in TNBC patients, suggesting a tumor-suppressive role of GPER in TNBC [37,38]. Shen et al. reported a negative correlation between AR and GPER in TNBC patient samples [109]. Further study is needed to elucidate the role of GPER, and other non-nuclear receptors, in clinical TNBC.

### 7.5. Endocrine Therapy for Patients with TNBC

Currently, cytotoxic chemotherapy is the only standardized treatment option for TNBC; however, AR-positive TNBC reportedly shows less chemotherapy responsiveness and a lower pathologic complete response rate after neoadjuvant treatment [12,114]. These findings indicate the need for a chemo-free alternative for AR-positive TNBC, or the possibility of sensitizing AR-positive TNBC for chemotherapy by modulating AR.

Androgen-inhibiting drugs such as bicalutamide, enzalutamide, and abiraterone acetate are expected to block the tumor-promoting effect of androgens (Figure 5), improving the prognosis of patients with LAR tumors. Grellety et al. reported that immunohistochemically defined apocrine features identified abiraterone acetate-responders in TNBC [115]. There have been many clinical trials (including ongoing ones) for androgen-targeting therapy in TNBC [5,12]. Some of them are on combined androgen-inhibiting therapy and other molecular-targeting therapy, such as palbociclib, ribociclib (CDK4/6), pembrolizumab (PD-1), and taselisib (PI3Kα inhibitor) [12].

Some clinical trials targeting ER-β to treat TNBC exist. The drugs estimated are toremifene or anastrozole (ClinicalTrials.Gov Identifier: NCT02089854), E2 (NCT03941730, ongoing), and tamoxifen (NCT02062489, ongoing).

Given the promising experimental results indicating the effect of ER-β to sensitize AR-positive TNBC for enzalutamide [105], clinical trials targeting both receptors is desired.

## 8. Materials and Methods 

A systematic literature search was performed on the PubMed database up to April 2021 using the keywords: triple-negative breast cancer, androgen receptor, estrogen receptor-β, and G-protein-coupled estrogen receptor. A manual search was also performed for the references listed in the obtained articles.

Pathological materials in Figure 4 were obtained from a patient included in the study approved by the ethics committee of Toho University Faculty of Medicine (A19079_A18116, 25 March 2020).

## 9. Conclusions

The role of estrogens and androgens in TNBC pathobiology has been intensively and extensively studied in preclinical and clinical settings. Despite the presence of controversy, promising results are accumulating regarding the importance of these hormones as therapeutic targets in TNBC. As shown here, these hormones show constitutive similarity among themselves and their receptors, and also show similarity in signaling mechanisms and therapeutic strategies. Further, recent experimental studies suggested the effect of combined androgen and estrogen-targeting therapy. Considering the coexistence of estrogens and androgens, or co-expression of ER-β and AR in a clinical setting, a comprehensive study considering both hormones is desired to optimize the treatment of TNBC.

## Figures and Tables

**Table 1:** Representative exogenous substances that exert agonistic or antagonistic effects on ER-β or GPER.

Effects	ER-β	GPER
Agonists	Isoflavone Daidzein Genistein Flavanone Liquiritigenin Prinaberel (ERB-041) Diarylpropionitrile (DPN) WAY200070	G1
Antagonists	PHTPP	G15 G36
